# Efficacy of first-line immunization combined with antiangiogenesis treatment and chemotherapy for the treatment of tongue cancer: A case report

**DOI:** 10.1097/MD.0000000000034660

**Published:** 2023-09-22

**Authors:** Limin Zhao, Yongmin Liu, Chunhong Chen, Lili Lv, Boran Xu, Lihua Su, Feng Gao

**Affiliations:** a Department of Oncology, Heilongjiang Provincial Land Reclamation General Hospital, Harbin, China.

**Keywords:** anlotinib, immune checkpoint inhibitors, tongue cancer, toripalimab

## Abstract

**Background:**

There is currently no uniform and effective treatment for patients with locally advanced oral cancer who cannot tolerate surgery or radiotherapy. The prognosis of oral cancer patients with lymph node metastasis is very poor, but the clinical treatment of such patients faces certain challenges.

**Patients and methods:**

Case 1 was a 59-year-old patient with tongue cancer (cT _3_ N _x_ M _0_ G _2_) who refused radiotherapy because of a history of leukoderma. After evaluation of disease condition, a 4-drug combination therapy of toripalimab + anlotinib + nabpaclitaxel + carboplatin was administered. Case 2 was a 55-year-old patient with tongue cancer (cT _3_ N _2_ M _0_ G _1_) who could not receive radiotherapy because of a medical history of cervicofacial burns. After disease evaluation, toripalimab + anlotinib + docetaxel + carboplatin combination therapy was administered.

**Case summary:**

Both patients did not experience any adverse reactions during treatment and achieved a complete response after 2 cycles of treatment. Their progression-free survival is currently 6 and 8 months, respectively, and they are in sustained remission.

**Conclusion:**

Currently, the efficacy of immune checkpoint inhibitors targeting programmed death-1 as a first-line treatment of inoperable and non-radiatable locally advanced oral cancer is unknown. Here, we describe 2 cases of locally advanced oral cancer treated with first-line immune checkpoint inhibitors in combination with targeted therapy and chemotherapy. This approach was successful in these patients, but a larger sample size is required to verify our findings.

## 1. Introduction

Tongue cancer is the most common oral malignancy worldwide. Approximately 25% of patients have developed cervical lymph node metastasis at the time of discovery, which seriously affects prognosis.^[1]^ Patients with locally advanced head and neck squamous cell carcinoma that have relapsed following surgery and/or chemotherapy have very poor prognoses, with a median survival of 10 months and a 2-year survival rate of <20%.^[2]^ According to the 2022 CSCO guidelines, the recommended first-line treatment modality is radiotherapy combined with cisplatin for locally advanced oral cancer patients who are ineligible for surgery. However, the guidelines do not provide a reference for patients ineligible for radiotherapy.^[3]^ Head and neck squamous cell carcinoma tumors are highly inflammatory with lymphocytic infiltration and strong programmed death ligand 1 (PD-L1) expression on tumor cells and cells within the tumor microenvironment (TME). Preclinical studies have shown that blocking the interaction between programmed death-1 (PD-1) and its ligand PD-L1 increases activation of cytotoxic T cells and inhibits tumor growth. Additionally, the TME plays important roles in promoting tumor cell proliferation, invasion, and local and distant metastasis of tongue cancer. These findings suggest that new treatment methods for tongue cancer require a combination of anticancer and TME-targeting drugs.^[4,5]^ Toripalimab is a recombinant humanized anti-PD-1 monoclonal antibody used to treat various cancers that binds to PD-1 and prevents association with its ligands PD-L1 and PD-L2.^[6]^ In December 2018, the results of a Phase 2 clinical trial and safety data from multiple clinical studies led to the approval of toripalimab for the treatment of unresectable or metastatic melanoma, following the failure of prior systemic therapy in China. Anlotinib is a small-molecule multi-target tyrosine kinase inhibitor that can effectively inhibit kinases such as VEGFR, PDGFR, FGFR, and c-Kit. This drug has antiangiogenic and tumor growth inhibitory effects, showing efficacy in solid tumors such as non-small cell lung cancer, soft tissue sarcoma, and medullary thyroid carcinoma.^[7]^ Immunization combined with antiangiogenesis therapies can alter the TME, while combination with chemotherapy can play a synergistic role. Currently, immunotherapy combined with targeted therapy and chemotherapy has become an area of exploration in the antitumor therapy field.

## 2. Case presentation

### 2.1. Case 1

**Figure 1.**
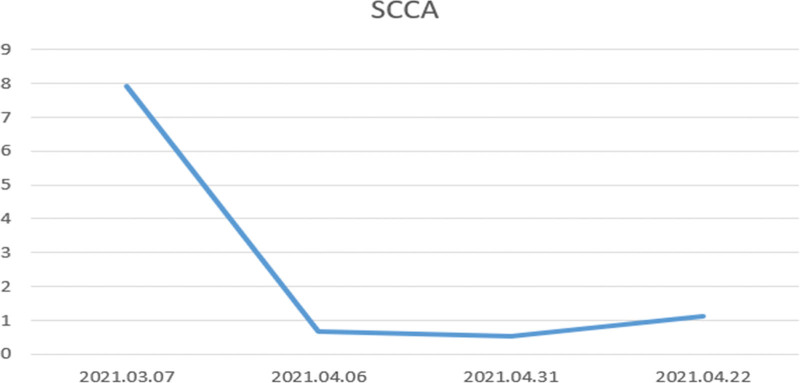
Treatment-emergent SCCA curve. March 2021, 07 SCCA: 7.9 1 ng/mL; SCCA Reference: 0 001–2.5 ng/mL.

Ding XX, female, 59 years old. In May 2020, the patient inadvertently found a mass at the lateral margin of the right side of her tongue, but was unbothered by it. In January 2021, the patient was found to have tumor enlargement and mild pain that affected eating, causing her to visit our hospital in March 2021. She had a history of hypertension for more than 10 years and denied habitual smoking and alcohol use. She had an ECOG score of 0, NRS scoreof1, and SCCA level of 7.91 ng/mL (Fig. 1).

**Figure 2.**
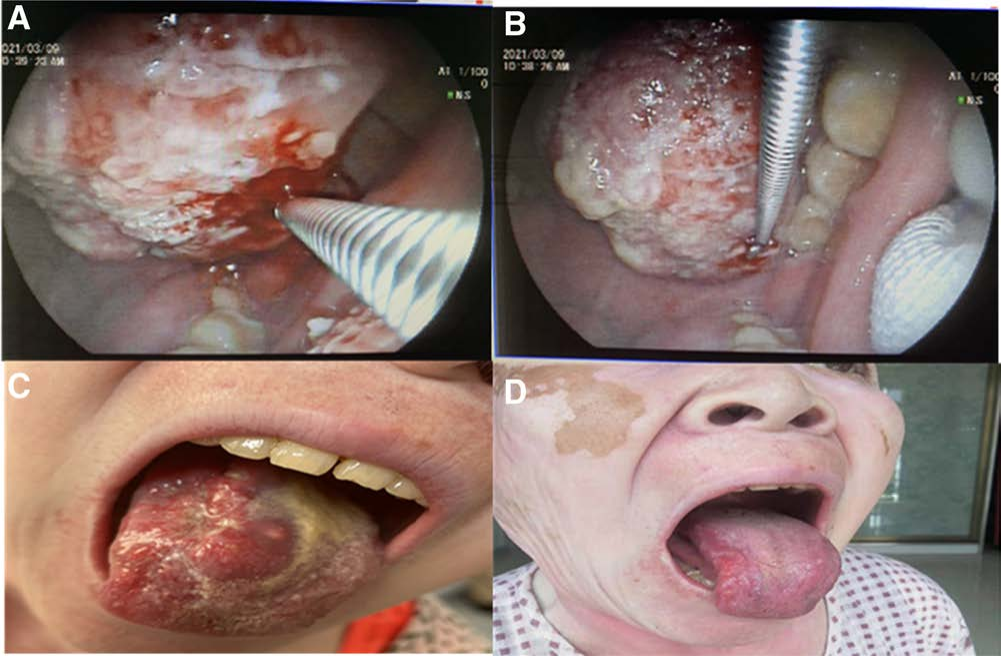
(A, B) Biopsy of tongue lesions. (C) A solid tumor lesion was observed on the right side of tongue before treatment. (D) The solid tumor nearly fully disappeared after two cycles of treatment.

After admission, a systemic examination indicated no distant metastasis. She was pathologically diagnosed with squamous cell carcinoma, but a PD-L1test was not performed. A clinical bed diagnosis indicated tongue squamous cell carcinoma (cT _3_ N _x_ M _0_ G _2_). Because the patient had a history of vitiligo, concurrent chemoradiotherapy was recommended. The patient herself did not agree to radiotherapy, sotoripalimab 240 mg was given orally on day 1, anlotinib 10 mg on days 1 to 14, as well as albumin paclitaxel 300 mg andcarboplatin 400 mg as part of a 4-drug combination therapy after a comprehensive evaluation. The patient’s clinical symptoms were significantly relieved 3 days after treatment, and a complete response was noted after 2 cycles. Following 4 cycles of maintenance treatment with toripalimab in combination with anlotinib, no adverse effects occurred and the patient currently shows no disease progression (Fig. 2).

### 2.2. Case 2

**Figure 3.**
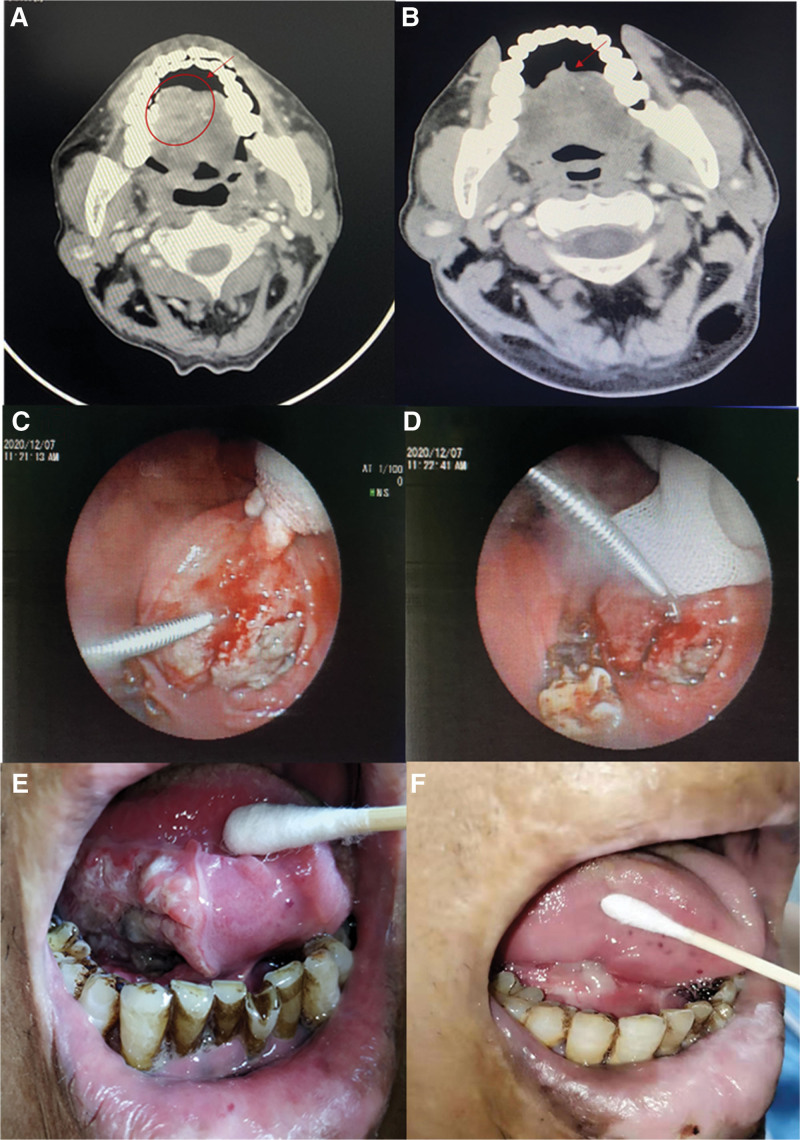
(A) Pretreatment images. (B) Images after two cycles of treatment. (C, D) Biopsy of tongue lesions. (E) Image of the solid lesion pretreatment. (F) Image of the solid lesion posttreatment.

Zou XX, male, 55 years old. In October 2020, the patient developed oral pain after eating. A local pathological biopsy of the floor of the mouth and base of the tongue suggested squamous epithelial hyperplasia with atypia and hyperkeratosis, and the tumor was to be excluded. He presented to our hospital in December 2020. The patient had a history of hypertension for more than 2 months, irregularly used oral antihypertensive drugs, and denied habitual smoking and alcohol use. He had a history of burns. He had an ECOG score of 0, NRS score of 1. Relevant examinations were completed after admission, and he was pathologically diagnosed with squamous cell carcinoma with cervical lymph node metastasis. Clinical diagnosis suggested squamous cell carcinoma of the tongue (cT _3_ N _2_ M _0_ G _1_ stage IVA). Because the patient was not suitable for surgery or radiotherapy, toripalimab 240 mg was administered orally on day 1, anlotinib 10 mg on days 1 to 14, as well as docetaxel 100 mg, carboplatin 400 mg as part of a 4-drug combination therapy. The patient’s clinical discomfort symptoms were significantly relieved 3 days after treatment, and 2 cycles led to a complete response. After 6 cycles of chemotherapy, toripalimab combined with anlotinib was administered. Thus far, 8 cycles have been completed without any adverse reactions. The lesions were in sustained remission (Fig. 3).

## 3. Discussion

Anlotinib, a small-molecule tyrosine kinase inhibitor, combined with toripalimab, a PD-1 inhibitor, can efficiently synergize to effectively promote chemokine release, induce T cell infiltration and activation, reduce the proportion of regulatory T cells, regulate the M1/M2 macrophage balance, and alter the TME. These effects collectively support better penetration of chemotherapeutic drugs into tumor cells, increase plasma drug concentrations, and enhance antitumor effects. The poor prognosis and clinical outcomes associated with tongue cancer are reportedly mainly caused by tumor resistance to chemotherapy. However, the mechanism behind this is not well understood. Head and neck malignancies are generally treated locally with surgery and chemoradiotherapy, but effective treatment options are urgently needed for patients who cannot tolerate surgery and radiotherapy or are reluctant to undergo such treatments. Immunotherapy with immune checkpoint inhibitors has achieved great success in the last decade. However, because of primary resistance to immunosuppressive agents, acquired resistance has emerged in some patients and single-agent immunosuppressive agents have limited efficacy.^[8]^ Neovascularization in the presence of hypoxia or specific genetic changes can occur in tumors that directly promote tumor growth and metastasis and can also support an immunosuppressive TME. antiangiogenic drugs can enhance the effect of immunotherapy by activating immune CD8 + T cells, reversing the immunosuppression caused by VEGF and promoting tumor vascular normalization. Immunosuppressive agents can simultaneously promote antiangiogenic effect by activating effector T cells, leading to vascular normalization and enhanced infiltration and killing functions of T cells. Therefore, immunotherapy and antiangiogenic drugs can form a positive feedback loop and synergize with each other.^[9]^ antiangiogenic drugs tend to normalize tumor vessels, improve the TME, increase the concentration of chemotherapeutic drugs in tumors, and alter hypoxia to improve the efficacy of chemotherapy drugs. In Case 1, the patient refused radiotherapy because of her history of vitiligo. Combined with the abovementioned theoretical basis, the patient was given an anti-PD-1 drug combined with anlotinib and chemotherapy. After 2 cycles of this combination treatment, the lesion achieved complete remission. Her current progression-free survival is 6 months. Akhter et al^[10]^ found that oral cancer patients with cervical lymph node metastases had a worse prognosis than those without lymph node metastases, and patients with metastasis to the cervical lymph nodes were more likely to develop distant metastases. The patient in Case 2 was unable to receive radiotherapy because of his history of cervicofacial burns. He was newly diagnosed with cervical lymph node metastasis and treated with anti-PD-1 therapy combined with anlotinib, as well as maintenance therapy with anti-PD-1 combined with anlotinib after 6 cycles of chemotherapy. Currently, the patient has sustained remission and 8 months progression-free survival. These 2 cases suggest that the new 3-pronged regimen combination of immunization with antiangiogenesis treatment and chemotherapy for tongue cancer showed clinical effects only after 1 cycle of treatment, with no obvious adverse reactions. Therefore, this regimen can be used for patients who cannot undergo surgery or do not show clinical benefit from surgery or radiotherapy. For further analysis, the patients should be subsequently observed for their overall survival. Some limitations of this study are that these 2 patients did not undergo genetic testing, nor was their immune marker status predicted. Furthermore, a stratification factor analysis should be performed with a larger sample size using next generation sequencing testing to determine if they have genetic variants that make them more suitable for antiangiogenesis therapies.

## Author contributions

**Conceptualization:** Limin Zhao.

**Data curation:** Limin Zhao, Boran Xu, Lihua Su.

**Formal analysis:** Yongmin Liu.

**Supervision:** Feng Gao.

**Validation:** Feng Gao.

**Writing – original draft:** Chunhong Chen, Lili Lv.

**Writing – review & editing:** Limin Zhao.
